# Effects of Melatonin on the Defense to Acute Hypoxia in Newborn Lambs

**DOI:** 10.3389/fendo.2019.00433

**Published:** 2019-07-12

**Authors:** Felipe A. Beñaldo, Aníbal J. Llanos, Claudio Araya-Quijada, Auristela Rojas, Alejandro Gonzalez-Candia, Emilio A. Herrera, Germán Ebensperger, Gertrudis Cabello, Guillermo J. Valenzuela, María Serón-Ferré

**Affiliations:** ^1^Programa de Fisiopatología, ICBM, Facultad de Medicina, Universidad de Chile, Santiago, Chile; ^2^International Center for Andean Studies (INCAS), Universidad de Chile, Santiago, Chile; ^3^Departamento de Biología, Facultad de Ciencias, Universidad de Tarapacá, Arica, Chile; ^4^Department of Women's Health, Arrowhead Regional Medical Center, San Bernardino, CA, United States

**Keywords:** melatonin, neonate, acute hypoxia, heart, adrenal, lung

## Abstract

Neonatal lambs, as other neonates, have physiologically a very low plasma melatonin concentration throughout 24 h. Previously, we found that melatonin given to neonates daily for 5 days decreased heart weight and changed plasma cortisol and gene expression in the adrenal and heart. Whether these changes could compromise the responses to life challenges is unknown. Therefore, firstly, we studied acute effects of melatonin on the defense mechanisms to acute hypoxia in the neonate. Eleven lambs, 2 weeks old, were instrumented and subjected to an episode of acute isocapnic hypoxia, consisting of four 30 min periods: normoxia (room air), normoxia after an i.v. bolus of melatonin (0.27 mg kg^−1^, *n* = 6) or vehicle (ethanol 1:10 NaCl 0.9%, *n* = 5), hypoxia (PaO_2_: 30 ± 2 mmHg), and recovery (room air). Mean pulmonary and systemic blood pressures, heart rate, and cardiac output were measured, and systemic and pulmonary vascular resistance and stroke volume were calculated. Blood samples were taken every 30 min to measure plasma norepinephrine, cortisol, glucose, triglycerides, and redox markers (8-isoprostane and FRAP). Melatonin blunted the increase of pulmonary vascular resistance triggered by hypoxia, markedly exacerbated the heart rate response, decreased heart stroke volume, and lessened the magnitude of the increase of plasmatic norepinephrine and cortisol levels induced by hypoxia. No changes were observed in pulmonary blood pressure, systemic blood pressures and resistance, cardiac output, glucose, triglyceride plasma concentrations, or redox markers. Melatonin had no effect on cardiovascular, endocrine, or metabolic variables, under normoxia. Secondly, we examined whether acute melatonin administration under normoxia could have an effect in gene expression on the adrenal, lung, and heart. Lambs received a bolus of vehicle or melatonin and were euthanized 30 min later to collect tissues. We found that melatonin affected expression of the immediate early genes *egr1* in adrenal, *ctgf* in lung, and *nr3c1*, the glucocorticoid receptor, in adrenal and heart. We speculate that these early gene responses may contribute to the observed alterations of the newborn defense mechanisms to hypoxia. This could be particularly important since the use of melatonin is proposed for several diseases in the neonatal period in humans.

## Introduction

Neonatal lambs, like human and other neonates, have low endogenous levels of plasma melatonin through 24 h (1–3). This is a rather unique situation, in which newborns are missing the physiological role of the nocturnal elevation of plasma melatonin to which fetuses and adults are exposed. Melatonin acts through diverse mechanisms (membrane receptors, interaction with orphan nuclear receptors, proteins, or as an antioxidant) and influence a diversity of physiological functions including cardiovascular, endocrine, and metabolic, among others (4, 5). Importantly, melatonin has antioxidant properties ameliorating perinatal hypoxic brain damage (6, 7). Nevertheless, there is little information regarding the effects of melatonin on other organ systems in the newborn.

Previously, we investigated the effects of melatonin in normal term newborn lambs by imposing a high-amplitude melatonin rhythm for 5 days (8). We found that this treatment resulted in a decreased heart weight, changes in cortisol and other plasma variables, changes of gene expression of clock genes and selected functional genes in the adrenal gland and heart. From this experimental design, we could not distinguish whether some of these responses were elicited acutely by melatonin. Furthermore, we could not discern the ability of these organs to respond to real-life challenges. Hypoxia is a well-known stressor for the newborn, which may be experienced acutely as well as chronically. Acute hypoxia elicits a prompt and complex integrated defense response involving the cardiovascular, respiratory, and endocrine systems, allowing, initially, an appropriate oxygen supply to tissues in response to a sudden reduction of blood PO_2_ (9, 10). In the fetus, an infusion of melatonin modulates the cardiovascular defense to acute hypoxia (11), whereas in the neonate, no data are available. Therefore, in the present study, we investigated the effects of a single bolus of melatonin on cardiovascular, endocrine, and metabolic responses to acute hypoxia in 2-week-old lambs. As mentioned, at this age the response to hypoxia has been well-characterized by us (10, 12) and other researchers (9) and lambs have not established the endogenous rhythm of melatonin yet (1). Continuing the investigation previously done, we assessed whether melatonin administered acutely can trigger gene responses in adrenals, lung, and heart in normoxia. We determined early gene expression (*egr1, ctgf*), glucocorticoid receptor (*nr3c1*), and clock genes (*per1, bmal1, cry1*) in the adrenal, lung, and heart in newborn sheep. With the acute i.v. administration of melatonin, we expect to find changes in the variables measured, as we found changes in several variables after 5 days of oral melatonin treatment.

## Materials and Methods

The Ethics Committee of Faculty of Medicine, University of Chile, approved all the experimental protocols (CBA #1000 FMUCH). Animal care, maintenance, procedures, and experimentation were performed in accordance with the Guide for the Care and Use of Laboratory Animals published by the US National Institutes of Health (NIH Publication No. 85-23, revised 1996) and adhere to American Physiological Society's Guiding Principles in the Care and Use of Animals.

### Animals

Eleven newborn lambs aged 2 weeks, gestated, born, and raised at Lluta Research Station (Faculty of Medicine, University of Chile, near-sea-level, in the Region of Arica and Parinacota, Chile), were randomly allocated into two groups: five lambs (two females and three males) to the control group (receiving vehicle) and six (two females and four males) to the melatonin group. Under general anesthesia, the lambs were instrumented in the pulmonary (Swan Ganz) and femoral artery/vein (polyvinyl catheters) (10). Lambs were subjected to two protocols. In protocol 1, 3 days after surgery, newborns were exposed to an episode of acute hypoxia (see below). After 30 min of normoxia, the lambs received a 1-ml bolus of 0.27 mg kg^−1^ of melatonin i.v. Melatonin solution was prepared by dissolving 100 mg of melatonin (Sigma-Aldrich, Quimica Limitada, Santiago, Chile) in 10 ml of ethanol and then diluted 1–10 in 0.9% NaCl. Control lambs received 1 ml of vehicle. Experiments were performed around noon. In protocol 2, the day after the hypoxia experiment, lambs were given a bolus of melatonin or vehicle at 14:00 h and euthanized 30 min after with sodium thiopentone 100 mg kg^−1^ i.v., (Tiopental; Laboratorio Biosano, Santiago, Chile). Body weights at euthanasia were 5.8 ± 0.9 kg and 5.9 ± 0.4 kg for the control and melatonin group, respectively.

### Surgical Preparation

All surgical procedures were performed under aseptic conditions as described previously (10). Briefly, at 14 days of age, lambs were instrumented under general anesthesia with ketamine, 10 mg kg^−1^ i.m., (Ketostop; Drag Pharma-Invectec, Santiago, Chile), xylazine, 0.1–0.5 mg kg^−1^ i.m., (Xilazina 2%, Laboratorio Centrovet, Santiago, Chile), and atropine (0.04 mg kg^−1^ i.m. (Atropina Sulfato; Laboratorio Chile, Santiago, Chile), with additional local infiltration of 2% lidocaine (Dimecaina; Laboratorio Beta, Santiago, Chile). Polyvinyl catheters (1.2 mm internal diameter) were placed into the descending aorta and inferior vena cava. A Swan-Ganz catheter (Swan-Ganz 5 French, Edwards Lifesciences LLC, Irvine, CA, USA) was placed in the pulmonary artery. All catheters were filled with heparin solution (1,000 IU ml^−1^ in 0.9% NaCl), exteriorized, and kept in a cloth pouch sewn onto the skin. Oxytetracycline, 20 mg kg^−1^, i.m., (Liquamicina LA, Pfizer, Chile), and sodium metamizole 0.1 mg kg^−1^, s.c., (Metamizol sódico, Laboratorio Chile, Chile) were given immediately after surgery and the following 2 postoperative days.

#### Protocol 1

Cardiovascular, endocrine, and metabolic response to acute isocapnic hypoxia.

**Figure 1:**
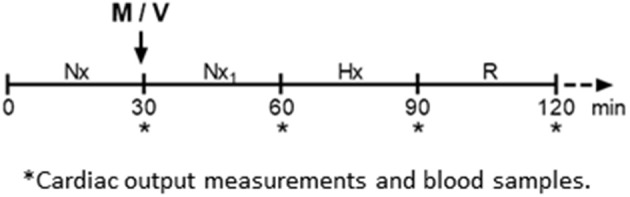
Protocol 1 description. Lambs were instrumented 3 days before the experiment. The day of the experiment, lambs were exposed to four sequential 30 min intervals of: normoxia (Nx, breathing room air), normoxia_1_ (Nx_1_) after an i.v., bolus of 0.17 mg kg^−1^ melatonin (M) or vehicle (V), hypoxia (Hx, PaO_2=_ 30 ± 2 mmHg) followed by recovery (R) under room air. – –Lambs returned to their mothers until next day (protocol 2). *Time of cardiac output measurements and blood sample collection. Heart rate and pulmonary and systemic arterial blood pressure were measured from 0 to 120 min.

Three days after surgery, the lambs were subjected to an episode of acute isocapnic hypoxia. The room temperature was set at 25°C (thermoneutral temperature as determined in previous studies, 12). The hypoxia protocol (Figure 1) consisted of four 30 min periods: normoxia (breathing room air), normoxia_1_ after an i.v., bolus of melatonin (0.17 mg kg^−1^) or vehicle (ethanol 1:10 NaCl 0.9%), hypoxia (PaO_2_: ~30 ± 2 mmHg), followed by recovery under room air. Hypoxia was induced by passing about 20 L min^−1^ of 10% O_2_ and 2–3% CO_2_ in N_2_ through a transparent polyethylene bag loosely tied over the animal's head (10). The newborn catheters were attached to pressure transducers and a data acquisition system connected to a computer (Powerlab/8SP System and Chart v4.1.2 Software; AD Instruments, New South Wales, Australia) recording continuously pulmonary and systemic pressures and heart rate. Additionally, every 15 min, we determined the cardiac output (CO) by thermodilution, injecting 3 ml of chilled (4°C) 0.9% NaCl into the pulmonary artery *via* the Swan-Ganz catheter connected to a cardiac output computer (COM-2 model; Baxter, Edwards Critical-Care Division, Irvine, CA) (12, 13). Blood gases, PaO_2_, PaCO_2_, and pH, were measured at fixed intervals during the protocol (12). Blood samples were drawn at the end of each 30 min interval to measure plasma melatonin concentration, catecholamines, cortisol, glucose, triglycerides, 8-isoprostane, and ferric reducing ability of plasma (FRAP). After recovery, lambs were returned to their mothers.

#### Protocol 2

**Figure 2:**
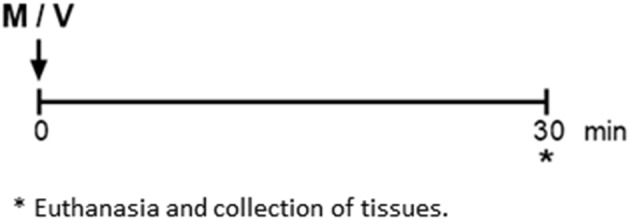
Protocol 2 description. The day after the hypoxia experiment, under room air, lambs received a bolus of 0.17 mg kg^−1^ melatonin (M) or vehicle (V) via the femoral catheter. *Lambs were euthanized, and heart, lung, and adrenals were collected to measure gene expression.

Acute gene expression response to melatonin in normoxia (Figure 2).

Newborns received a bolus of melatonin or vehicle at 14:00 h and were euthanized 30 min after with sodium thiopentone 100 mg kg^−1^ i.v., (Tiopental; Laboratorio Biosano, Santiago, Chile). Adrenal glands, lung, and heart were dissected, weighed, and stored in liquid nitrogen for molecular biology studies (12).

### Plasma Assays

Melatonin, 8-isoprostane, triglycerides, and glucose concentration in plasma were measured using the following kits according to the manufacturer's recommendations. Melatonin: Elisa, MyBioSource, San Diego, CA, 92195-3308, USA; 8-isoprostane: Elisa, Cayman Chemical, Ann Arbor, MI, 48108, USA; glucose and triglycerides: Valtek Diagnostics, Santiago, Chile. FRAP was measured as described previously (14). Plasma cortisol, epinephrine, norepinephrine, and dopamine were measured by high-performance liquid chromatography in a commercial laboratory (Barnafi Krause Diagnostica, Santiago, Chile).

### qPCR

Heart, lung, and adrenal samples (about 100 mg) were homogenized in TRIzol. The RNA fraction was subjected to DNase treatment using the SV Total RNA Isolation System (PROMEGA, Madison, WI). RNA obtained was resuspended in nuclease-free water and the absorbance was measured at 260 and 280 nm. The ratio of 260 to 280 was 1.9 to 2.05. The RNA was stored at −20°C. RNA reverse transcription was performed from approximately 2 μg of RNA with 100 ng of random primers and 200 U M-MLV RT (200 U/μl) in a final volume of 20 μl. Gene expression was measured by quantitative reverse transcription polymerase chain reaction (PCR) as previously reported (8). Primers reported in the literature were used to measure *egr1, ctgf* (15), *per1, bmal1, cry1*, glucocorticoid receptor (*nr3c1*), *gapdh*, and *rplp0* (8). Reverse transcription PCR conditions were 65°C for 5 min, 4°C for 5 min, 37°C for 2 min, 25°C for 10 min, 37°C for 50 min, and 70°C for 15 min. Assays were performed in a StepOne thermal cycler from Applied Biosystems, CA, USA, using 5x Hot FIREPol Eva Green HRM Mix (Solis BioDyne, Riia, Tartu, Estonia) following the manufacturer's instructions. Non-template controls were included in every PCR reaction and three complementary DNA pools were included to assess inter-assay variability. The threshold cycle of each sample and the internal control was interpolated in the respective standard curve. Gene expression was measured as the ratio to *gapdh* for adrenal and lung, and *rplp0* for the heart.

### Statistical Analysis

For cardiovascular, metabolic, and endocrine variables, data were expressed as fold change vs. normoxia, means ± standard error (SEM). Changes within each group were analyzed by one-way ANOVA for repeated measures followed by the *post hoc* Dunnett test. Groups were compared by two-way ANOVA followed by the *post hoc* Newman–Keuls test (16). Gene expression data were analyzed by Student *t* test or Mann–Whitney non-parametric test. Analysis were performed using Prism 6.01, GraphPad Software, La Jolla, CA. For all comparisons, differences were considered statistically significant when *P* ≤ 0.05.

## Results

### Protocol 1

**Figure 3:**
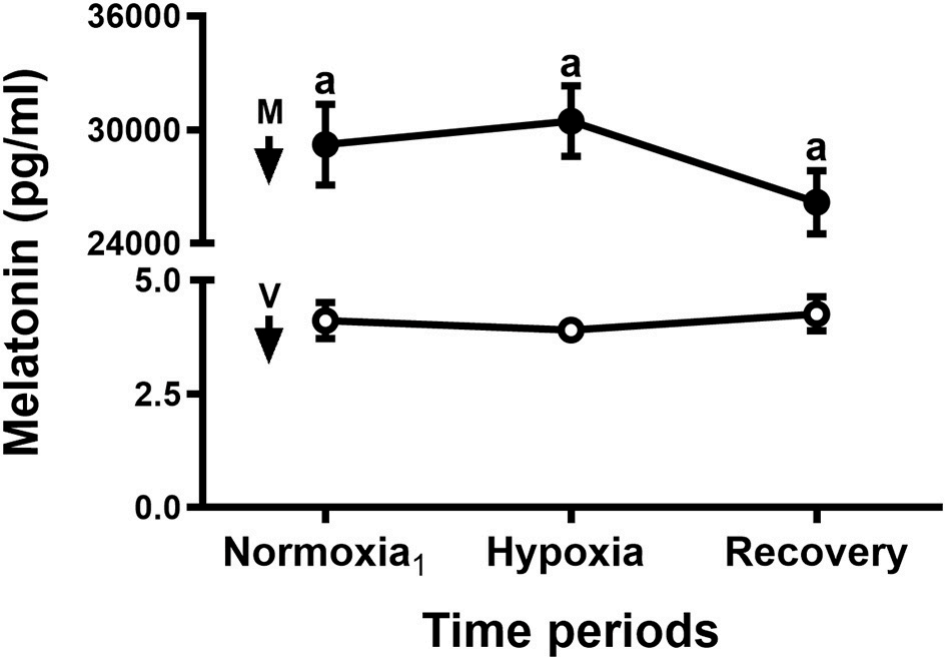
Plasma melatonin concentration during an episode of acute hypoxia. Thirty minutes before taking the normoxia_1_ blood sample, five newborn lambs received an i.v., bolus of vehicle (1 ml 10% ethanol-saline, white circles) and six i.v., bolus of melatonin (0.27 mg kg^−1^ in 1 ml of vehicle, black circles). Arrows indicate time of bolus (V, vehicle; M, melatonin). Values are means ± SEM. Significant differences, *P* < 0.05: a, melatonin group vs. vehicle group in the same time periods.

**Figure 4:**
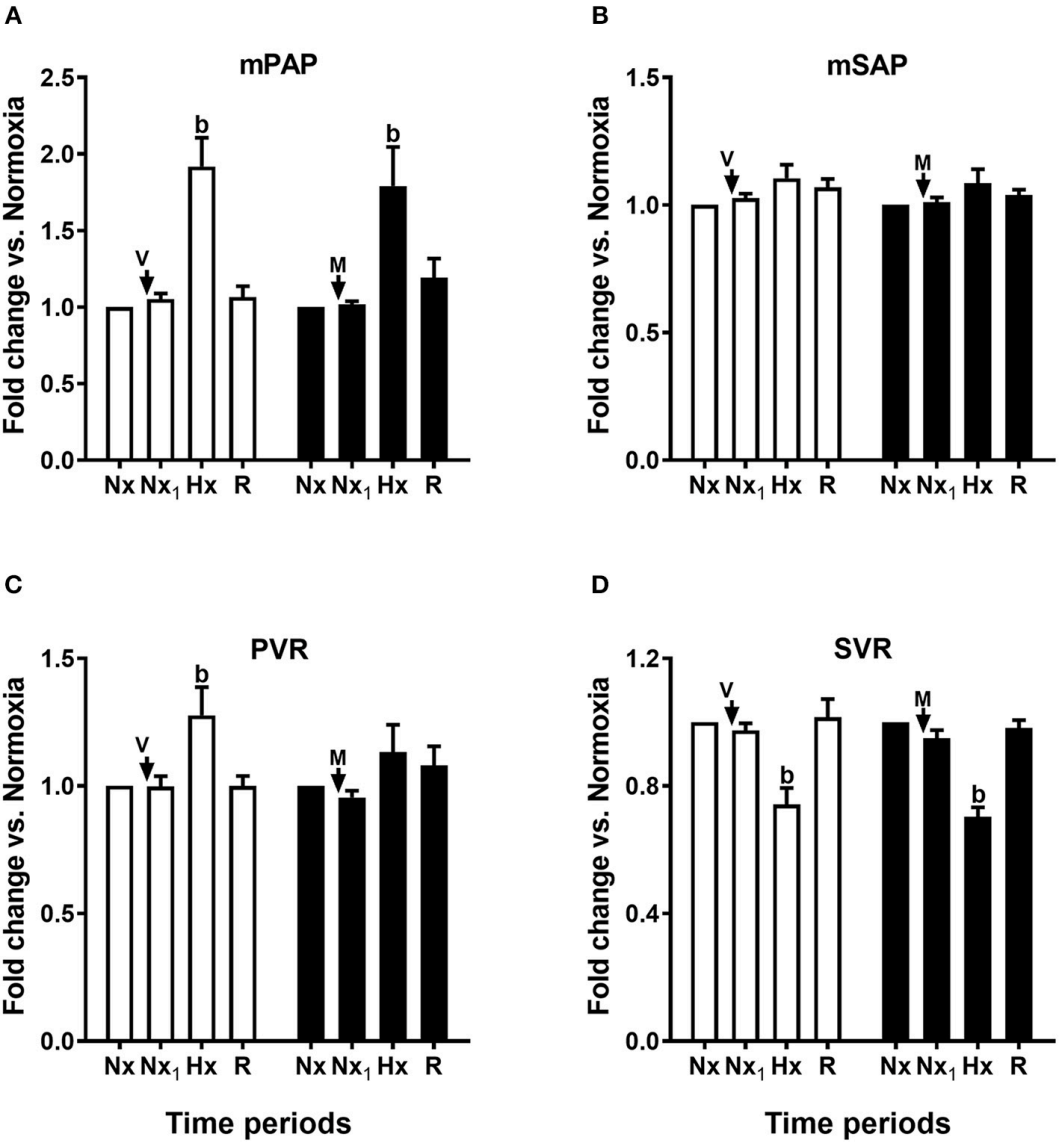
Acute effects of melatonin on pulmonary and systemic circulation responses to an episode of acute hypoxia. Mean pulmonary arterial pressure (mPAP, **A**), systemic arterial pressure (mSAP, **B**), pulmonary vascular resistance (PVR, **C**), and systemic vascular resistance (SVR, **D**). Control newborn lambs (vehicle, V, white bars) and newborn lambs treated with an i.v., bolus of melatonin (0.27 mg kg^−1^; M, black bars). Arrows indicate time of bolus. Nx: normoxia, Nx_1_: normoxia after bolus, Hx: hypoxia, R: recovery. Values are means ± SEM. Significant differences, *P* < 0.05: b, hypoxia vs. other time periods in the same group.

**Figure 5:**
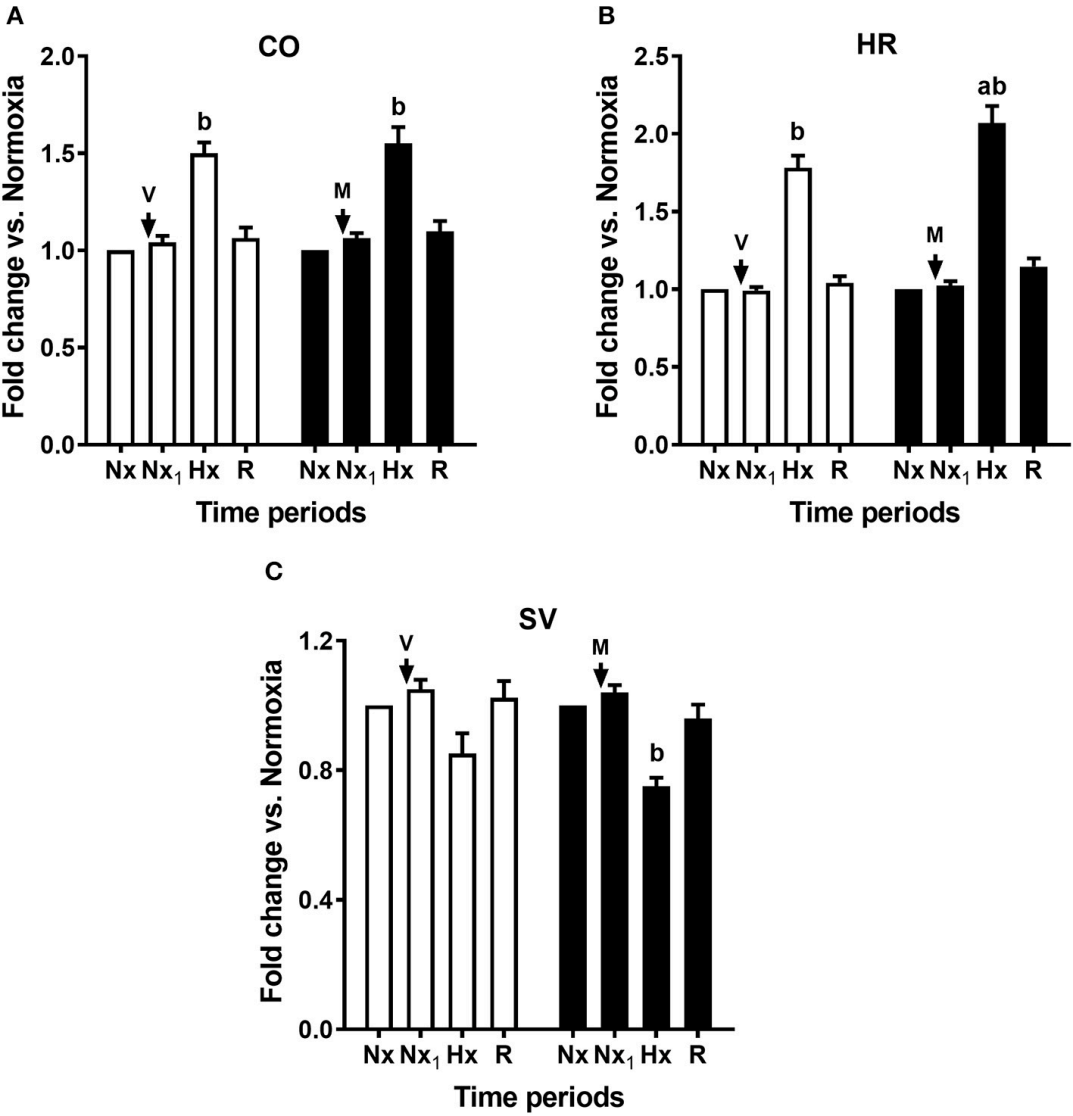
Acute effects of melatonin on cardiac output, heart rate, and stroke volume responses to an episode of acute hypoxia. Cardiac output (CO, **A**), heart rate (HR, **B**), and stroke volume (SV, **C**). Control newborn lambs (vehicle, V, white bars) and newborn lambs treated with an i.v., bolus of melatonin (0.27 mg kg^−1^, M, black bars). Arrows indicate time of bolus. Nx, normoxia; Nx_1_, normoxia after bolus; Hx, hypoxia; R, recovery. Values are means ± SEM. Significant differences, *P* < 0.05: a, melatonin group vs. control group in the same time period; b, hypoxia vs. other time periods in the same group.

**Figure 6:**
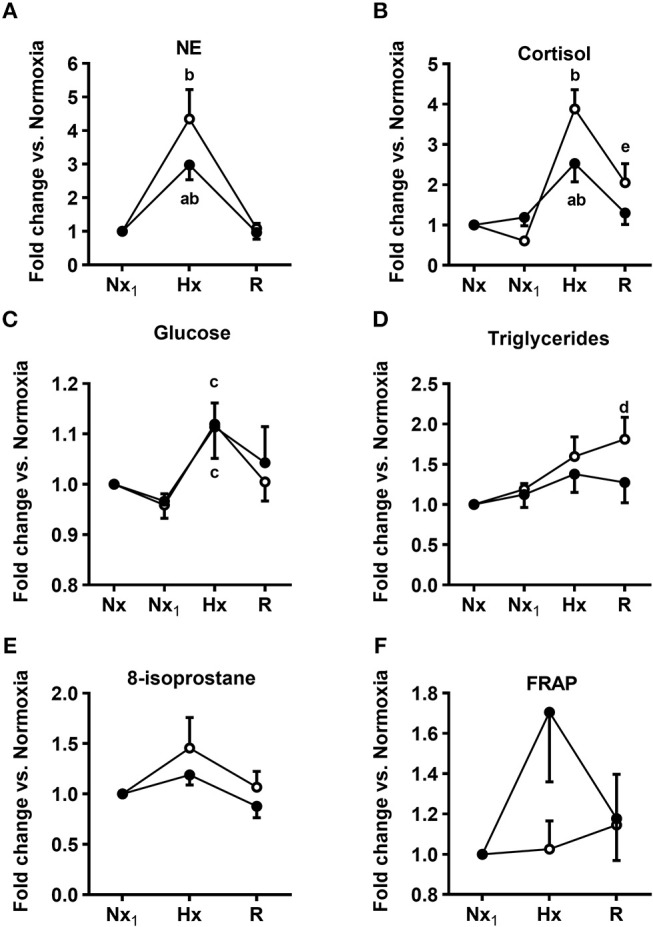
Acute effect of melatonin on the response of plasma endocrine, metabolic variables, and Reactive oxygen species (ROS) markers during an episode of acute hypoxia. Norepinephrine (NE, **A**), cortisol **(B)**, glucose **(C)**, triglyceride **(D)**, 8-isoprostane **(E)**, and FRAP **(F)** plasma concentrations. Control newborn lambs receiving vehicle (white circles) and newborn lambs receiving an i.v., bolus of melatonin (0.27 mg kg^−1^, black circles) 30 min before the first sampling interval. Nx, normoxia; Nx_1_, normoxia after bolus; Hx, hypoxia; R, recovery (room air). Values are means ± SEM. Significant differences, *P* < 0.05: a, melatonin group vs. control group in the same period; b, hypoxia vs. other time periods in the same group; c, hypoxia vs. Nx_1_; d, recovery vs. Nx in the same group; e, recovery vs. other time periods in the same group.

The control neonates, during normoxia and hypoxia, had similar pH, blood gases, cardiovascular (10), and endocrine (8) variables to newborns of previous protocols. Control newborns showed very low levels of melatonin, as expected, which remained constant throughout the experiment (Figure 3). Administration of an i.v. melatonin bolus resulted in very elevated plasma melatonin levels (about 5,000-fold) compared to lambs receiving vehicle (Figure 3). Melatonin concentration remained elevated during the whole hypoxia protocol and recovery period. Hypoxia had no effect on plasma melatonin concentration in control newborns (Figure 3). Neither melatonin nor vehicle changed blood gases during normoxia (Table 1). Moreover, there were no cardiovascular, endocrine, or metabolic responses to melatonin or vehicle during normoxia (Figures 4–6).

**Table 1:** Arterial pH and blood gases during an episode of acute hypoxia in neonatal lambs.

**Variable**	**Treatment**	**Normoxia**	**Normoxia_**1**_**	**Hypoxia**	**Recovery**
pH	Vehicle	7.455 ± 0.011	7.447 ± 0.009	7.445 ± 0.015	7.458 ± 0.013
	Melatonin	7.453 ± 0.007	7.433 ± 0.009	7.428 ± 0.016	7.420 ± 0.015
PaO_2_ (mmHg)	Vehicle	91.8 ± 4.6	89.5 ± 4.0	29.5 ± 0.4b	97.6 ± 3.2
	Melatonin	97.8 ± 2.2	94.8 ± 2.2	29.6 ± 0.6b	96.1 ± 1.5
PaCO_2_ (mmHg)	Vehicle	41.2 ± 1.5	38.7 ± 2.0	40.3 ± 2.7	37.9 ± 1.1
	Melatonin	36.2 ± 0.7	33.9 ± 2.0	34.1 ± 1.8	34.3 ± 0.7

(Means ± SEM). Normoxia_**1**_: normoxia after bolus of melatonin or vehicle. Melatonin, n = 6; vehicle, n = 5.

*b = p < 0.05 vs. other time periods in the same group. See text for details*.

In contrast, we found that melatonin altered the cardiovascular and endocrine response to hypoxia. As shown in Table 1, both control and treated newborns reached similar PO_2_ during hypoxia, while maintaining PCO_2_ at the level seen during normoxia (isocapnic hypoxia) (12). As seen in Figures 4, 5, control newborns showed the known cardiopulmonary response to hypoxia characterized by increased mean PAP and pulmonary arterial resistance (white bars, Figures 4A,C), no changes in mean SAP, with decreased in systemic vascular resistance (white bars, Figures 4B,D). Further, there was an increase in cardiac output and increases in heart rate without changes in stroke volume (white bars, Figures 5A–C). Melatonin treatment significantly blunted the PVR responses to hypoxia (Figure 4C) but exacerbated the HR response to hypoxia (Figure 5B) and decreased the stroke volume (Figure 5C). The responses in mPAP, mSAP, SVR (Figures 4A,B,D), and CO (Figure 5A) were not affected by melatonin. Neither melatonin nor vehicle affected cardiovascular variables under normoxia (Figures 4, 5).

Melatonin had no effect on plasma endocrine and metabolic variables under normoxia (Figures 6A–F). In control newborns, hypoxia elicited a sharp increase in plasma norepinephrine, cortisol, and glucose concentration (white circles, Figures 6A–C). Triglycerides had a late rise in the recovery period (white circles, Figure 6D), while 8-isoprostane and FRAP did not change during the experiment (white circles, Figures 6E,F). In the melatonin-treated neonates, there was a blunted increase in plasma norepinephrine and cortisol concentration in response to hypoxia (black circles, Figures 6A,B), while the glucose levels in plasma augmented like those in the controls (black circles, Figure 6C). There were no changes in plasma concentrations of triglycerides, 8-isoprostane, and FRAP in the melatonin group during hypoxia (black circles, Figures 6D–F). Dopamine and epinephrine plasma concentrations did not change either in controls or in melatonin-treated neonates, neither in normoxia nor in hypoxia (data not shown).

### Protocol 2

**Figure 7:**
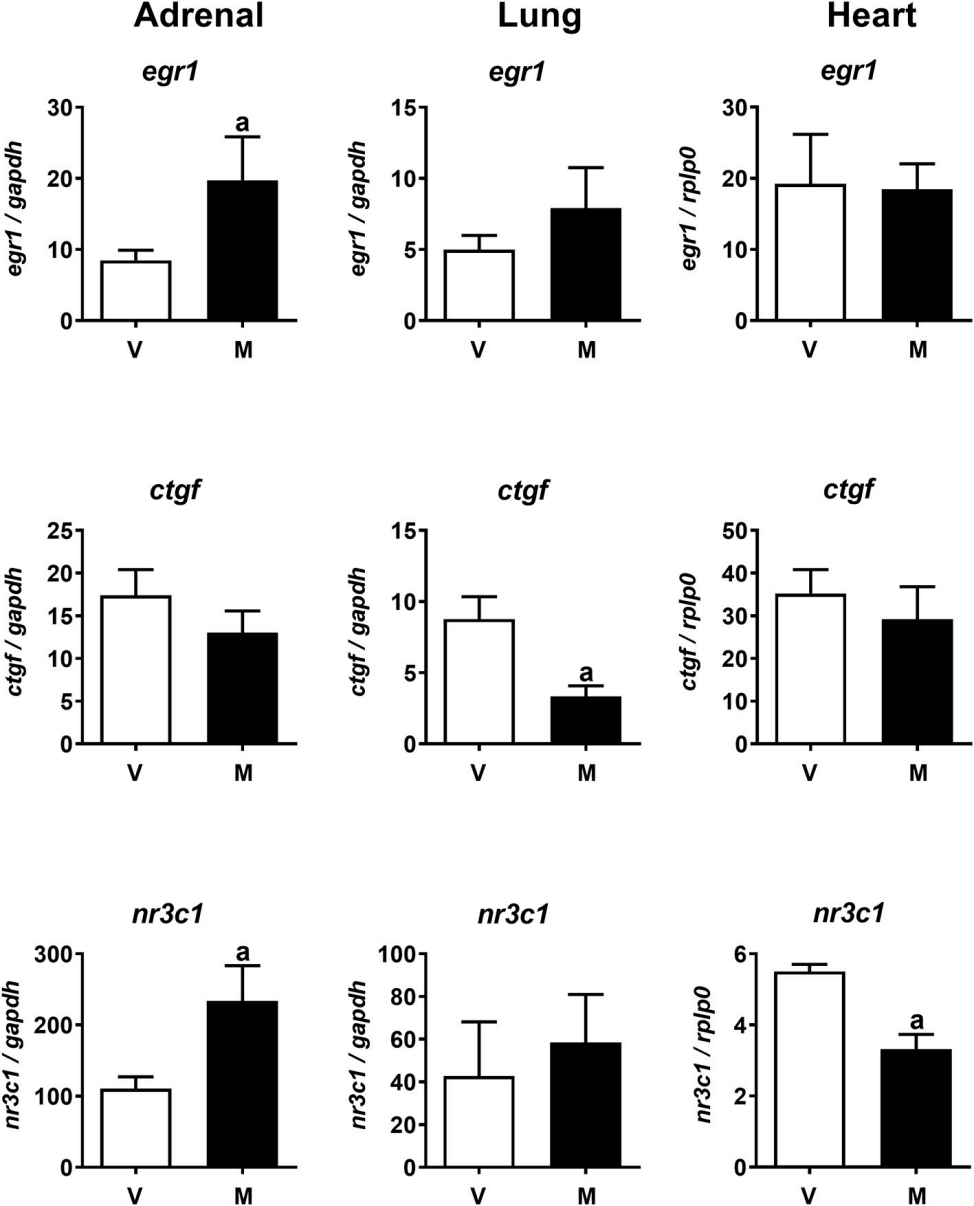
Acute effects of melatonin on IEG expression during normoxia in adrenal, lung, and heart. *egr1*: **(Top)**, *ctgf* : **(Middle)**, *nr3c1*: **(Bottom)**. V, vehicle group; M, melatonin group. Values are means ± SEM. Significant differences, *P* < 0.05: a, melatonin group vs. vehicle group.

The lack of cardiovascular, endocrine, or metabolic effects of melatonin under normoxia contrasts with the fast responses seen in hypoxia. We wondered whether melatonin may have rapid effects at the tissue level that were not detected in the previous experiments. We explored whether 30 min exposure *in vivo* to a high dose of melatonin without the hypoxia challenge affected expression of some immediate early genes (IEGs) in the adrenal, lung, and heart (left ventricle, LV). We measured the expression of *egr1, ctgf*, glucocorticoid receptor (*nr3c1*), *per1, bmal1*, and *cry1* in these tissues. The panels of Figure 7 show that gene expression responses to melatonin in normoxia are gene and tissue specific. Melatonin increases *egr1* expression in the adrenal but had no effect on *egr1* expression in lung and heart (Figure 7, top, black bars). Likewise, melatonin induced a decrease in *ctgf* in the lung and not in the adrenal or heart (Figure 7, central, black bars). Regarding the glucocorticoid receptor (*nr3c1*), melatonin had opposite effects on the adrenal and heart, increasing its expression in the former while decreasing it on the latter. No effect was observed in the lung (Figure 7, bottom, black bars). A tendency to increase the clock genes *bmal1* (*P* = 0.09) and *cry1* (*P* = 0.08) was observed in adrenal and only for *cry1* in the heart (*P* = 0.09); no effects of melatonin on *per1* expression were detected (data not shown).

## Discussion

In this study, we found that acute melatonin administration modified the cardiovascular and endocrine components of the newborn lamb's defense to acute hypoxia. Melatonin blunted the increase in pulmonary vascular resistance, exacerbated the heart rate response, and induced a decrease in heart stroke volume. Further, melatonin decreased the responses of norepinephrine and of cortisol plasma concentration to hypoxia. Melatonin had no effect on cardiovascular and endocrine variables under normoxia, despite the elevated levels of plasma melatonin achieved. However, we found acute changes in gene expression under normoxia, showing early effects of melatonin on the adrenal, lung, and heart that could prime the altered cardiovascular and endocrine responses to hypoxia. The beneficial use of melatonin to ameliorate perinatal hypoxic brain damage (6, 7) should be balanced with the impairment of the defense mechanism to acute hypoxia elicited by this molecule.

Hypoxia is a challenge that may be experienced with high incidence by the newborn, acutely as in the neonatal respiratory distress syndrome (17) or chronically as in bronchopulmonary dysplasia (18) or at high altitude (19). In this study, we explored the effect of melatonin on the neonatal defense to acute hypoxia.

Acute hypoxia triggers defensive mechanisms that preserve oxygen homeostasis and cellular functions in the whole organism. The neonatal responses to acute hypoxia (9, 10) have not been extensively studied as have the fetal cardiovascular responses to this stressor (20–22). As seen in our vehicle-treated lambs, acute hypoxia increased heart rate, cardiac output, pulmonary arterial pressure, and pulmonary vascular resistance in relation to basal values while decreasing systemic vascular resistance and maintaining systemic blood pressure. Other studies show similar results to our control neonates for these variables (10). In these, hypoxia resulted in slow falls in femoral blood flow and vascular resistance, suggesting that responses to hypoxia were initiated by a weak chemoreflex function (10). Moreover, as shown by Sidi et al. (9), heart and brain blood flows increased, and carcass flow was unchanged, whereas skin, gastrointestinal tract, and kidney flow fall, while adrenal blood flow augments importantly (23). The result is a blood flow redistribution in the neonate toward the vital circulations, such as the brain, heart, and adrenals, the so-called “brain sparing effect,” described in detail in the fetus (22). Concurrent with these cardiovascular changes, in acute hypoxia, there is an increase in plasma cortisol and norepinephrine (Llanos et al, unpublished data, and present study) and in ANP and BNP (24), among other hormones. In addition, metabolic changes take place, as exemplified by a sharp increase in plasma glucose (25).

Acute melatonin treatment decreased pulmonary vascular resistance, consistent with evidences *in vivo* that chronic melatonin treatment induced vasodilation in pulmonary arteries of chronically hypoxic neonatal sheep (26) and *in vitro* adult rat pulmonary vessels (27, 28). A particular property of pulmonary vasculature is that it contracts in response to hypoxia, contrary to vessels of the heart, brain, or adrenals, which dilate in hypoxia. The vascular smooth muscle cells of the pulmonary arterioles respond directly to low O_2_ partial pressure (PO_2_), with a pulmonary vasoconstriction even with no neural or endocrine influences (29). The low PO_2_ decreases voltage-gated K^+^ channels activity, resulting in pulmonary smooth muscle cells depolarization. This elicits a major increase in intracellular Ca^2+^ concentration, resulting in vasoconstriction (29, 30). Compensatory mechanisms induced by hypoxia in the pulmonary vessels may be changes in NO availability, by eNOS stimulation, activating sGC and triggering a vasodilation cascade (31). Which of these mechanisms were modified by melatonin to lower pulmonary resistance induced by hypoxia is unknown. Melatonin acts through mechanisms such as membrane receptors (MT1 and MT2), direct action on ionic channel activation, antioxidant actions, etc., depending on the type of cell and physiological context (4). Accordingly, there are evidences that melatonin could decrease intracellular Ca^2+^ concentration and NO production in some tissues. For example, in PC12 cells and in rat hippocampus slices, melatonin blocks voltage-sensitive Ca^2+^ channels, reducing intracellular Ca^2+^ concentration. This effect is seen with concentrations of melatonin in the nanomolar range and is not mediated by melatonin receptors (32). Furthermore, in mesenteric arteries, melatonin exerts vasodilation by direct action on the BKCa^2+^ channel and activating eNOS through receptor-mediated actions (33). Moreover, melatonin significantly suppresses ROS-induced inhibition of NO through its ability to scavenge hydroxyl radicals in human umbilical arteries (34). Additionally, in favor of a melatonin effect mediated by increases in NO is the experiment of Thakor et al. (11) showing that melatonin effects on fetal cardiovascular and metabolic responses to hypoxia were inhibited by using a NO clamp, i.e., suppressing NO production. Some of these mechanisms could participate during hypoxia to decrease the pulmonary vascular resistance in our lambs. The melatonin receptors MT1 and MT2 are present in various vascular beds, including lung, heart, and adrenals of the sheep newborn (Seron-Ferre, unpublished results). In most vascular territories, the MT1 receptor mediates vasoconstrictor and MT2 vasodilator effects of melatonin (35). An exception is the pig coronary artery, in which melatonin triggers vasoconstriction acting through the MT2 receptor. In these arteries, melatonin augmented PDE5 catalytic action, by phosphorylation, reducing cGMP and NO-induced relaxation (36). In adult humans, acute melatonin augments forearm, maintains cerebral, and decreases renal blood flows, indicating different effects on some vascular territories (37). The different results on blood flow and resistances in the organs throughout the body show the melatonin pleiotropic and unexpected outcomes on these vital functions.

The second cardiovascular effect of melatonin was the increased heart rate in addition to that already induced by hypoxia. The heart rate response to hypoxia is controlled by a reflex initiated in the carotid body chemoreceptors. Thus, low PO_2_ depolarizes the glomus cells (38), triggering action potentials through the sinus nerve of Hering and glossopharyngeal nerve that synapses in the *nucleus tractus solitarius* (NTS) located in the brainstem. The NTS indirectly modulates the activity of sympathetic neurons in regions of the central nervous system regulating the autonomic control of the heart, producing tachycardia (39). Of note, melatonin enhanced the carotid chemoreceptor response to hypoxia *via* melatonin receptors in the rat carotid body, increasing intracellular Ca^2+^ concentration in the glomus cells, carotid afferent nerve activity, respiratory frequency, and ventilation during hypoxia (40). The increase in glomus cell intracellular Ca^2+^ concentration induced by melatonin is opposite to what is observed in some smooth vascular cells in which the intracellular Ca^2+^ concentration decreased. This conundrum can be explained by the myriad of actions that melatonin exerts in different tissues. An exacerbation in the chemoreflex could lead to the increase in heart rate in the neonatal lambs treated with melatonin in relation to controls.

The third cardiovascular effect of melatonin was the reduction in stroke volume of the heart. This cardiovascular variable is calculated by dividing the cardiac output by the heart rate. Since the heart rate is augmented importantly in the melatonin-treated neonates, the stroke volume decreased. Worth mentioning, melatonin possesses anti-adrenergic effects in isolated rat papillary muscle that could reduce contractility and stroke volume (41). Additionally, melatonin inhibits voltage-sensitive Ca^2+^, decreasing intracellular Ca^2+^ concentration that could reduce heart contractility and therefore stroke volume (32). The exacerbated tachycardia and the reduction in stroke volume are undesirable alterations of the defense mechanism in hypoxic neonatal melatonin-treated lambs.

The defense to acute hypoxia in the newborn also involves endocrine and metabolic responses. Hypoxia induces an increase in sympathetic tone reflected as increased plasma norepinephrine that was blunted by melatonin. This observation agrees with previous work in the fetal sheep (11) and the finding that suppression of fetal melatonin by exposing pregnant ewes to constant light increases plasma norepinephrine in their newborns (13). Moreover, exposing newborn lambs to a high-amplitude rhythm of melatonin reduces plasma norepinephrine (8). *In vivo*, studies in the adult human and rats show acute inhibitory effects of melatonin in the modulation of a variety of functions of the sympathetic system (42, 43). Melatonin receptors are present in the sheep adrenal gland (44) and in areas of the brain that may be involved in sympathetic nervous system control in human, sheep, rat, and newborn pigs (45–47). Importantly, norepinephrine can contract the small pulmonary arteries of the newborn sheep (10). Thus, norepinephrine-diminished response to hypoxia possibly contributed to the decreased pulmonary vasoconstriction found in the melatonin-treated neonates during hypoxia.

Another important response to acute hypoxia is an increase in plasma cortisol levels. Our experiments show that melatonin lessened this response. Plasma cortisol levels reflect adrenal cortisol secretion, which is regulated by Adrenocorticotropic hormone (ACTH), adrenal innervation, and the endogenous adrenal circadian clock (48). Isocapnic hypoxia increases plasma ACTH in chronically catheterized rats (49), and whether this occurred in the current experiments is not known. Nevertheless, there is extensive evidence showing direct actions of melatonin on the adrenal. The adrenal gland expresses an MT1 receptor, and melatonin *via* this receptor directly inhibits the stimulatory action of ACTH on the adult adrenal of human, non-human primates, rat (50–52), sheep (44), and monkey fetal adrenal (53). On the other hand, splanchnic nerve stimulation increased rat adrenal corticosterone release in the absence of an increase of ACTH and augmented adrenal clock gene *per1* expression (54). Clock genes are important in the adrenal response to ACTH as deletion of the clock genes inhibits cortisol response to ACTH (55, 56). Previously, we showed that daily treatment with melatonin inhibits adrenal clock gene oscillation in newborn lambs (8). Most likely, the acute blunting effect of melatonin on hypoxia-induced cortisol elevation in the newborn lambs combines direct effects on the adrenal and the effects on autonomic innervation just discussed.

The adaptive mechanisms of hypoxia include metabolic changes triggered by the decreased availability of oxygen. In general, metabolic changes have been better studied for chronic hypoxia, but at least the glucose metabolic response to acute hypoxia is well-known in newborn lambs. Plasma glucose concentration is the balance between secretion to and uptake from the plasma compartments. Glycogenolysis and gluconeogenesis stimulated by adrenalin and cortisol, respectively, and uptake by tissues due to insulin effects are the main factors in this regulation. Melatonin is known to decrease β-cell insulin release (57), and in humans, melatonin impairs the glucose tolerance test, prolonging a high glucose plasma concentration (58). We have also found that treatment of newborn lambs with daily doses of melatonin resulted in elevated glucose levels (8). We speculate that, in the present study, decreased insulin, combined with the decrease in sympathetic tone and cortisol, may explain the lack of melatonin effect on the hypoxia-triggered plasma glucose elevation. Two other findings pertain to reduced metabolic effects of acute hypoxia in the newborns of the present study. We did not see an increase in plasma triglycerides levels, akin to findings during acute hypoxia in humans (59). In addition, we did not see changes in plasma oxidative stress markers in response to acute hypoxia. *In vitro* studies show that, at the cell level, decreased availability of oxygen brings a short-lived increase in superoxide (about 10 min), due mainly to changes in mitochondrial function. (60). However, *in vivo* physiological compensation by cardiovascular mechanisms may suffice to deliver enough oxygen to tissues. Alternatively, only a few territories were affected, not enough for the changes to be detected in plasma. Under these conditions, we did not find effects of melatonin. Favoring the former interpretation, total oxygen consumption is maintained under acute hypoxia of the same magnitude in newborn lambs (10). Important effects of melatonin on plasma and tissue markers of oxidative stress have been detected in pulmonary tissue of chronically hypoxic high-altitude newborns (14, 26), pointing to a difference between acute and chronic effects of both hypoxia and melatonin.

Adrenal, lung, and heart are central to the aspects of cardiovascular and endocrine functions tested in this study. However, melatonin administration had no effect under normoxia on the cardiovascular and endocrine variables derived from these organs. We wondered whether melatonin elicits changes at the cellular level that could prepare the cells in these organs for further responses. Indeed, we found that 30 min *in vivo* exposure to melatonin under normoxia changed the expression of some IEGs in adrenal and lung. IEGs, also known as primary response genes, are defined as “sets of genes that respond in a short time (minutes) to cell-extrinsic and cell-intrinsic signals and do not require *de novo* protein synthesis for their expression” and are involved in a wide range of biological responses (61, 62). We measured expression of early growth response protein (*egr1*) and connective tissue growth factor (*ctgf*) that have been shown to respond quickly in lung (15) or *egr1* in heart (63). In addition, we measured the expression of the clock genes *per1, bmal1, cry1*, and the glucocorticoid receptor, *nr3c1* (nuclear receptor subfamily 3 group c member 1), that, in *in vitro* experimental protocols, were modified by melatonin in the adrenal gland (64, 65) and *pars tuberalis* (66). We found acute melatonin effects on gene expression in adrenal, lung, and heart; however, responses differed between tissues. Melatonin increased expression of the IEG *egr1* in the newborn adrenal gland but not in the heart or lung, whereas in the lung, melatonin decreased expression of *ctgf* and no changes were detected in the adrenal or heart. The lamb adrenal, lung, and heart contain a circadian oscillator. Previous work has shown that *in vitro* melatonin shifts clock genes in adult primate and rat fetal adrenal (64, 65). Moreover, daily melatonin treatment disrupts adrenal clock and alters the heart clock in lambs (8). In the present study, we found a tendency for increases in *bmal1* and *cry1* in the adrenal and in *cry1* in the heart of melatonin-treated newborns, consistent with acute effects of melatonin on the circadian clock. Another gene altered acutely by melatonin was the glucocorticoid receptor (*nr3c1*). In the adrenal, this receptor is involved in regulation of glucocorticoid synthesis, by intra-adrenal negative cortisol feedback (67), and in the heart, it participates in regulation of multiple genes (68). Additionally, mice with conditional disruption of glucocorticoid receptor in cardiomyocytes and vascular smooth muscle cells showed major changes in structural, functional, and biochemical maturation of the fetal heart, indicating that glucocorticoid signaling throughout this receptor is vital for a normal heart development and function (69). We wonder whether a decrease in the glucocorticoid gene expression with melatonin may have a functional outcome in the observed reduction of stroke volume during hypoxia, since a similar dose of melatonin that reduced glucocorticoid receptor was given to the neonates 30 min before the hypoxic insult.

In conclusion, the present data show acute effects of melatonin in the newborn at the cellular level and eventually undesirable alterations in organ systems participating in the defense strategy to a hypoxic challenge. These findings could be particularly relevant when considering the clinical use of melatonin in human neonates.

## Data Availability

All datasets generated for this study are included in the manuscript.

## Ethics Statement

The Ethics Committee of Faculty of Medicine, University of Chile approved all the experimental protocols (CBA #1000 FMUCH). Animal care, maintenance, procedures, and experimentation were performed in accordance with the Guide for the Care and Use of Laboratory Animals published by the US National Institutes of Health (NIH Publication No. 85-23, revised 1996) and adheres to American Physiological Society's Guiding Principles in the Care and Use of Animals.

## Author Contributions

FB, AL, GV, and MS-F conceived and designed the experiments. FB, AL, CA-Q, AR, GC, A-GC, EH, GV, and MS-F collected, analyzed, and interpreted the experimental data. FB, AL, and MS-F drafted the article. All authors revised it critically and approved the final version.

### Conflict of Interest Statement

The authors declare that the research was conducted in the absence of any commercial or financial relationships that could be construed as a potential conflict of interest.
